# Visceral-to-peripheral adiposity ratio: a critical determinant of sex and ethnic differences in cardiovascular risks among Asian Indians and African Creoles in Mauritius

**DOI:** 10.1038/s41366-024-01517-3

**Published:** 2024-04-13

**Authors:** Vinaysing Ramessur, Sadhna Hunma, Noorjehan Joonas, Bibi Nasreen Ramessur, Yves Schutz, Jean-Pierre Montani, Abdul G. Dulloo

**Affiliations:** 1 Obesity Research Unit, Biochemistry Dept., Central Health Laboratory, Victoria Hospital, Ministry of Health & Wellness, Plaines Wilhems, Mauritius; 2 Department of Endocrinology, Metabolism & Cardiovascular System, Faculty of Science & Medicine, University of Fribourg, Fribourg, Switzerland

**Keywords:** Preclinical research, Physiology

## Abstract

**Background/Aims:**

Coronary heart disease morbidity and mortality are higher in people of South Asian origin than in those of African origin. We investigated whether as young adults without diabetes, people in Mauritius of South Asian descent (Indians) would show a more adverse cardiovascular risk profile that those of predominantly African descent (Creoles), and whether this could be explained by ethnic differences in visceral adiposity or other fat distribution patterns.

**Methods:**

The study was conducted in 189 young non-physically active adults, with the following measurements conducted after an overnight fast: anthropometry (weight, height, waist circumference), whole-body and regional body composition by dual-energy x-ray absorptiometry, blood pressure, and blood assays for glycemic (glucose and HbA1c) and lipid profile (triglycerides and cholesterols).

**Results:**

The results indicate higher serum triglycerides and lower HDL cholesterol in men than in women, and in Indians than in Creoles (*p* < 0.001). No significant differences due to sex or ethnicity are observed in body mass index and waist circumference, but indices of visceral adiposity (visceral/android, visceral/subcutaneous) and visceral-to-peripheral adiposity ratio (visceral/gynoid, visceral/limb) were significantly higher in men than in women, and in Indians than in Creoles. The significant effects of sex and ethnicity on blood lipid profile were either completely abolished or reduced to a greater extent after adjusting for the ratio of visceral-to-peripheral adiposity than for visceral adiposity per se.

**Conclusions:**

In young adults in Mauritius, Indians show a more adverse pattern of body fat distribution and blood lipid risk profile than Creoles. Differences in their fat distribution patterns, however, only partially explain their differential atherogenic lipid risk profile, amid a greater impact of visceral-to-peripheral adiposity ratio than that of visceral adiposity per se on sex and ethnic differences in cardiovascular risks; the former possibly reflecting the ratio of hazardous (visceral) adiposity and protective (peripheral) superficial subcutaneous adiposity.

## Introduction

It is now recognized that people of certain race-ethnic groups experience a disproportionately greater burden of type 2 diabetes and cardiovascular disease, with the most compelling evidence deriving from studies conducted on first-generation migrants or their descendants living in Europe, North America, and in other diaspora countries. In particular, people with origins from countries of South Asia (Indian subcontinent) have a higher prevalence of type 2 diabetes, coronary heart disease, and cardiovascular mortality than Caucasians of European ancestry [1–6]. African-Americans show higher rates of diabetes, coronary heart disease, and stroke than white Americans of European descent [4, 7], while black people of African-Caribbean origins living in the UK have higher rates of diabetes and stroke than white British Europeans [5, 6]. Furthermore, while people with origins from South Asia and sub-Saharan Africa share higher risks for type 2 diabetes than people of European Caucasian origin [5–7], those of South Asian origins have higher risks for coronary heart disease than people of African-Caribbean origins [5, 8].

In Mauritius, an island nation whose multi-ethnic population comprises primarily those of South Asian (Indian) ancestry and of predominantly African ancestry (Creoles), national health surveys conducted since the mid-1980’s have also documented a high prevalence of type 2 diabetes and cardiovascular disease in both these ethnicities [9–12]. Notably, while the prevalence of type 2 diabetes was reported to be equally high both in Mauritian Indians and Creoles [9, 10] and to increase to similar extent in both ethnicities over the past decades [11], the Indians seem more prone to coronary heart disease and stroke than the Creoles [13, 14]. Among the major factors that could be involved in the greater predisposition of Mauritian Indians than Creoles to cardiovascular diseases, a less favorable cardiovascular risk profile (higher triglycerides and lower HDL cholesterol), has been reported among Indians [15–17], although this was not explained by ethnic differences in abdominal adiposity [15]. The latter, however, was assessed anthropometrically as waist-to-hip ratio, and hence may not accurately reflect central adiposity. Given evidence that proneness to store fat in visceral adipose tissue is an independent predictor for the development of atherogenic dyslipidemia and cardiovascular disease risks [18–22], the aims of the study reported here were to investigate:

(i) whether as young adults without diabetes, Mauritian Indians would show a less favorable cardiovascular risk profile and a more adverse body fat distribution pattern characterized by higher visceral adiposity compared to Creoles, and
(ii) the extent to which potential differences in visceral fat or other fat distribution patterns could explain differences due to sex and ethnicity in the cardiovascular risk profile of these young adults.

## Subjects and methods

### Participants and study design

Healthy young adult participants (*n* = 189) were recruited from the two main ethnic groups: those of South Asian (Indian) ancestry and those of predominantly African/Malagasy ancestry (Creoles); these two ethnicities constituting two-thirds and a-quarter of the island’s population, respectively [23]. They were eligible if they were men or women of 18–40 years of age, with relatively stable body weight (defined as <3% variation during the past 3 months), and non-physically active as defined by the Sedentary Behaviour Research Network [24]. Smokers, people who regularly consume alcoholic drinks, and women with menstrual irregularities, pregnant or breastfeeding women were excluded. The study was conducted in accordance to the guidelines of the Declaration of Helsinki, and was approved by the Ethics Committee of the Ministry of Health and Wellness, Republic of Mauritius (ethical approval reference code: MHC/CT/NETH/RAME); written informed consent was obtained from all participants.

### Anthropometry

Body weight was measured on an electronic weighing scale (Tanita Corporation, Tokyo), height was measured using a portable stadiometer (Tanita Leicester Height Measure, Leicester, UK), and waist circumference (WC) was measured at navel level using a non-stretchable tape, and according to the Standardization Reference Manual of Lohman et al. [25].

### Body composition and fat distribution

Whole-body composition was determined by dual-energy X-ray absorptiometry (DXA) using a HologicTM Horizon® QDR® WI System (Hologic Inc., Bedford, MA, USA), and according to guidelines for DXA procedures [26]. Scans were also analyzed to estimate the regional fat mass using the standardized regions specified by the manufacture for trunk, android, gynoid, and appendicular (limb) fat, as well as visceral adipose tissue mass (also referred to as visceral fat) using the Hologic Visceral Fat software. Abdominal subcutaneous fat (i.e., subcutaneous fat in the android region) was calculated as the difference between android fat and visceral fat. Peripheral adiposity refers to gynoid fat or appendicular (limb) fat, the latter being the sum of fat mass of the arms and legs.

### Blood assays and blood pressure

Resting blood pressure (systolic and diastolic) was measured by oscillometry using an OMRON® M2 automatic blood pressure monitor (OMRON Healthcare Ltd., Milton Keynes, UK), after which a blood sample was collected. HbA1c was measured on the same day on whole blood by HPLC (TosohG8, Tosoh Bioscience Inc., Tokyo, Japan). The other blood parameters were measured from plasma or serum (obtained by centrifugation and stored at −20 °C until later assays) using automated clinical analyzers (Abbott Architect c8000, Illinois, USA), namely plasma glucose and serum concentrations of triglycerides (TG), total cholesterol (Total-C) and HDL cholesterol (HDL-C). The serum value for LDL-cholesterol (LDL-C) was calculated using the Friedewald formula [27].

### Data analysis and statistics

Data analyses were performed using statistical software (STATISTIX version 8.0; Analytical Software, St Paul, Minnesota, USA), the figures were made using Graphpad Prism Software (version 9.3.1 for Windows, San Diego, CA, USA). The tabulated data are presented as Mean ± standard deviation (SD), and the data analyzed by two-factor analysis of variance and covariance to test for significance the effect of sex, ethnicity, and sex-ethnicity interaction. Because of its skewed distribution, the values of TG were logarithmically (log10) transformed to normalize the distribution prior to the application of statistical analyses. Linear model procedures were also applied for statistical comparisons of the two regression lines for equality of variance, slopes, and elevations (that is, y-intercepts). The analytical software for comparison of regression lines utilizes the analysis of covariance (ANCOVA). For all tests, significance was set at *p* < 0.05.

## Results

### Physical characteristics

The participants (*n* = 189) were young adults (mean/median age of ~26 years) with a large range of BMI varying between 14 and 37 kg/m^2^ (mean/median of 24.5 and 24 kg/m^2^, respectively). At the time of recruitment, all subjects were apparently healthy on the basis of questionnaire and interview. Nevertheless, upon performing their blood assays and taking their blood pressure, fasting blood glucose and HbA1c were found to be above limits of normality for 12 and 44% of the subjects, respectively, while about a-third of the subjects had high diastolic blood pressure (Supplementary Table S1). Among them, a few were diagnosed with overt diabetes (*n* = 2) or hypertension (*n* = 4). Their data are nonetheless included in the analysis as presented in the Tables and Figures here, as sensitivity analysis indicates that their omission had no impact on result outcomes and interpretations.

Their physical characteristics, according to sex and ethnicity, are presented in Table 1. No significant differences across groups/subgroups are observed for age, BMI, and WC. While men show higher stature than women by about 14 cm on average (1.74 vs 1.60 m, *p* < 0.001), analysis by ethnicity indicates that Indians were on average shorter than Creoles, with the difference in height reaching statistical significance in men (1.73 vs 1.78 m, *p* < 0.05) though not in women (1.60 vs 1.62 m). Analysis of body composition as fat mass index (FMI), fat-free mass index (FFMI), and appendicular soft lean mass index (ALMI) – the latter a proxy for skeletal muscle mass - indicate significant effects of sex and ethnicity for FFMI and ALMI, with values for Indians being lower than for Creoles. By contrast, FMI is observed to be higher in Indians than in Creoles in men, but not in women, with ANOVA indicating a significant effect of sex (*p* < 0.001), and not of ethnicity.

**Table 1:** Age, anthropometry, and body composition of subjects (*n* = 189) according to gender and ethnicity (Mean ± SD).

	Population sample *n* = 189			Men *n* = *73*		Women *n* = 116		ANOVA	
	Men *n* = 73	Women *n* = 116		Indian *n* = 52	Creole *n* = 21	Indian *n* = 71	Creole *n* = 45	Sex effect	Ethnic effect
Age (y)	26.6	25.9	ns	26.7	26.1	26.9	24.4	*ns*	*ns*
	±5.2	±5.0		±5.6	±4.4	±5.3	±4.0		
Height (m)	1.74	1.60	***	1.73	1.78	1.60	1.62	***	**
	±0.08	±0.07		±0.07	±0.07	±0.07	±0.06		
Weight (kg)	74.4	63.3	***	72.2	79.9	61.2	66.4	***	**
	±16.9	±12.6		±16.4	±17.3	±11.6	±13.6		
BMI (kg/m^2^)	24.4	24.6	ns	24.1	25.1	24.1	25.4	*ns*	*ns*
	± 5.0	±4.8		±5.2	± 4.7	±4.6	±5.0		
WC (cm)	89.4	86.5	ns	89.3	89.9	86.0	87.1	*ns*	*ns*
	±12.5	±10.5		±12.6	±12.3	±10.5	±10.6		
FFMI (kg/m^2^)	17.4	14.6	***	17.1	18.2	14.1	15.3	***	***
	±2.3	±2.0		± 2.3	±2.3	±1.8	±2.1		
ALMI (kg/m^2^)	7.91	6.15	***	7.73	8.36	5.90	6.59	***	***
	±1.20	±1.02		±1.12	±1.24	±0.89	±1.06		
FMI (kg/m^2^)	6.88	10.0	***	6.97	6.67	9.94	10.1	***	*ns*
	±2.94	±3.0		±3.08	±2.63	±2.97	±3.04		

Note that for ANOVA test, all the Sex x Ethnic interaction effects are not significant (results not shown).

*ns* not significant, *BMI* body mass index, *FFMI* fat-free mass index, *ALMI* appendicular lean mass index, *FMI* fat mass index.

**p < 0.01; ***p < 0.001.

### Body composition and fat distribution patterns

**Fig. 1:**
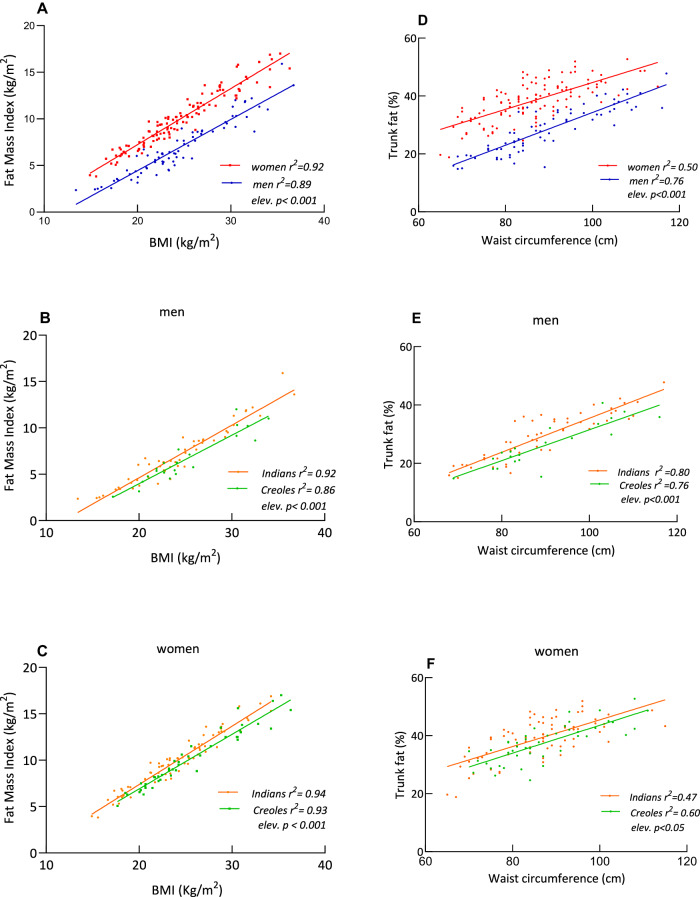
Relationship between adiposity and anthropometry. Plots of total body fat as Fat Mass Index (FMI) vs Body Mass Index (BMI) (left panels) and between trunk fat% vs waist circumference (right panels) according to sex (**A**, **D**) and ethnicity in men (**B**, **E**) and women (**C**, **F**). Within each panel, *elev.* refers to statistical significance in the elevation between the two regression lines, that is, in their *y*-intercepts.

**Fig. 2:**
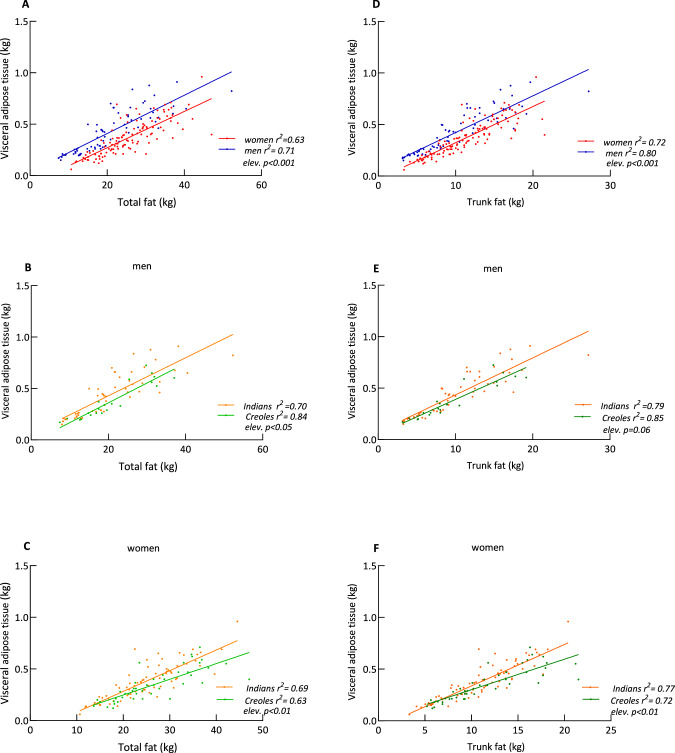
Relationship between adiposity compartments. Plots of visceral adipose tissue mass vs total fat mass (Left panels), and between visceral adipose tissue mass vs trunk fat mass (right panels) according to sex (**A**, **D**) and ethnicity in men (**B**, **E**) and women (**C**, **F**). Within each panel, *elev.* refers to statistical difference in the elevation between the two regression lines, that is, in their *y*-intercepts.

Application of linear regression analysis, however, indicates both sex and ethnic differences in the BMI-FMI relationship, as well as in the WC-trunk fat% relationship, as judged by statistically significant y-intercepts (elevations) when comparing sex and ethnicity within each sex (Fig. 1, A–F). For the same BMI (or for same WC), women show higher total FMI (and higher trunk fat%) than men (A, D), and within each sex, Indians show greater total FMI (and greater trunk fat%) than Creoles (B–F). Furthermore, sex and ethnic differences are also observed for regression plots of visceral adipose tissue (VAT) as a function of total body fat or trunk fat, as indicated by significant differences in the y-intercept for VAT vs total body fat (Fig. 2, A–C), and VAT vs trunk fat (D–F). These results indicate that for the same amount of total body fat or trunk fat, men show higher VAT than women (A, D), while Indians show higher values for VAT than Creoles, independently of sex (B, C, E, F).

The results of these regression analyses suggesting disproportionately higher visceral fat in men than in women, and in Indians than in Creoles, are also observed in some central adiposity indices, specifically in the ratios of visceral/android fat and visceral/subcutaneous fat in the abdominal region (Table 2), as well as in the indices of visceral-to-peripheral adiposity, namely in the ratios of visceral/gynoid fat and visceral/limb fat. These significant effects in adiposity indices contrast with a lack of statistical significance for the effects of sex and ethnicity on absolute values (kg) of central adiposity (android fat, visceral fat, abdominal subcutaneous fat), as well as after adjusting these measures of central adiposity for height (or height^2^) as covariate. Indeed, no significant correlations are observed between these measures of central adiposity and height (or height^2^) whether analyzed for the whole population sample or separately for men and women. Furthermore, while a significant sex effect (men > women), is also observed in the ratio of android/gynoid fat, no significant effect of ethnicity is observed for this adiposity index.

**Table 2:** Analysis of DXA-derived regional fat mass and adiposity indices according to sex and ethnicity (Mean ± SD).

.	Men		Women		ANOVA	
	Indian (*n* = 52)	Creole (*n* = 21)	Indian (*n* = 71)	Creole (*n* = 45)	*Sex* *effect*	*Ethnic* *effect*
**Central adiposity** (kg)						
Android	1.81	1.71	1.84	1.84	*ns*	*ns*
	*±1.01*	*±0.96*	*±0.73*	*±0.86*		
Visceral	0.44	0.38	0.39	0.34	*ns*	*ns*
	*±0.21*	*±0.17*	*±0.17*	*±0.15*		
Subcutaneous	1.36	1.33	1.45	1.496	*ns*	*ns*
	*±0.83*	*±0.80*	*±0.58*	*±0.74*		
**Peripheral adiposity** (kg)						
Gynoid	3.57	3.62	4.72	5.04	***	*ns*
	*±1.55*	*±1.20*	*±1.21*	*±1.31*		
Limb (appendicular)	9.48	10.2	13.0	13.8	***	*ns*
	*±4.38*	*±3.65*	*±3.62*	*±3.83*		
**Central adiposity indices** *(ratio)*						
Visceral/Android	0.27	0.24	0.21	0.19	***	**
	*±0.06*	*±0.05*	*±0.05*	*±0.04*		
Subcutaneous/Android	0.733	0.759	0.790	0.809	***	**
	*±0.061*	*±0.049*	*±0.047*	*±0.038*		
Visceral/Subcutaneous	0.373	0.320	0.270	0.239	***	**
	*±0.118*	*±0.091*	*±0.086*	*±0.060*		
**Central-to-peripheral adiposity indices** (ratio)						
Android/Gynoid	0.482	0.445	0.380	0.354	***	*ns*
	*±0.124*	*±0.119*	*±0.090*	*±0.095*		
Visceral/Gynoid	0.126	0.104	0.081	0.067	***	***
	*±0.036*	*±0.023*	*±0.031*	*±0.020*		
Subcutaneous/Gynoid	0.356	0.340	0.30	0.286	***	*ns*
	*±0.104*	*±0.106*	*±0.067*	*±0.081*		
Visceral/Limb	0.048	0.037	0.029	0.025	***	***
	*±0.015*	*±0.008*	*±0.010*	*±0.008*		
Subcutaneous/Limb	0.135	0.122	0.109	0.105	**	*ns*
	*±0.039*	*±0.038*	*±0.024*	*±0.030*		

For ANOVA test, all the *Sex x Ethnic interaction effects* are not significant (results not shown).

*ns* not significant.

**p < 0.01; ***p < 0.001.

### Cardiometabolic health

The results on cardiometabolic health markers are presented in Table 3. No significant effect of sex or ethnicity is observed for fasting blood glucose and HbA1c. By contrast, highly significant effects due to sex and ethnicity are observed for TG (men > women; Indians > Creoles) and HDL-C (men < women; Indians < Creoles). A significant effect of ethnicity (*p* < 0.05), but not of sex, is also observed for Total-C and LDL-C (Indians > Creoles). Furthermore, a highly significant effect of sex (*p* < 0.001) is observed for both systolic and diastolic blood pressure (men > women by about 14 and 6 mm Hg, respectively), but there is no significant effect due to ethnicity.

**Table 3:** Analysis of cardiometabolic health markers according to sex and ethnicity (Mean ± SD).

	Men		Women		ANOVA	
	Indian (*n* = 52)	Creole (*n* = 21)	Indian (*n* = 71)	Creole (*n* = 45)	*Sex effect*	*Ethnic effect*
*Glycemic profile*						
Glucose *(mmol/l)*	5.25	4.96	5.02	4.98	*ns*	*ns*
	*±1.02*	*±0.46*	*±1.04*	*±0.46*		
HbA1c *(%)*	5.76	5.50	5.65	5.62	*ns*	*ns*
	*±0.72*	*±0.46*	*±0.66*	*±0.44*		
*Lipid profile*						
Triglycerides (TG) *(mmol/l)*	1.83	1.11	0.98	0.75	***	***
	*±1.66*	*±0.92*	*± 0.55*	*±0.29*		
Cholesterol (C)						
HDL-C *(mmol/l)*	1.17	1.37	1.33	1.51	**	***
	*±0.21*	*±0.49*	*±0.27*	*±0.35*		
Total-C *(mmol/l)*	4.79	4.36	4.62	4.50	*NS*	*
	*±0.99*	*±0.72*	*±0.77*	*±0.79*		
LDL-C *(mmol/l)*	2.89	2.55	2.90	2.70	*NS*	*
	*±0.73*	*±0.60*	*±0.67*	*±0.73*		
*Blood pressure (BP)*						
Systolic BP *(mm Hg)*	117	121	104	106	***	*ns*
	*±12*	*±14*	*±12*	*±9*		
Diastolic BP *(mm Hg)*	81	81	75	74	***	*ns*
	*±9*	*±11*	*±9*	*±8*		

For ANOVA test, all the *Sex x Ethnic interaction effects* are not significant (results not shown).

*ns* not significant.

*p < 0.05; **p < 0.01; ***p < 0.001.

The results of covariance analysis in examining the extent to which any significant effect of sex or ethnicity for a given cardiometabolic health marker (i.e. unadjusted) is altered after controlling for various adiposity indices (as covariants) are presented in Table 4. The significant effects of sex on TG and HDL-C (unadjusted *p* < 0.001 and *p* < 0.01, respectively) are not altered after adjusting for any of parameters of central adiposity (visceral, abdominal subcutaneous, visceral/android; visceral/subcutaneous), or for peripheral adiposity (limb fat or gynoid fat). By contrast, they are completely abolished (ns) after adjusting for indices of visceral-to-peripheral adiposity (visceral/gynoid, visceral/limb), while only marginally reduced after adjusting for abdominal subcutaneous-to-peripheral adiposity. Furthermore, in relation to blood pressure (BP), the highly significant effect of sex on systolic BP is not altered after adjusting for any of these adiposity indices. By contrast, the strong significant effect sex on diastolic BP is completely abolished after adjusting for the ratio of visceral-to-peripheral adiposity, but only marginally reduced after adjusting for abdominal subcutaneous-to-peripheral adiposity ratio. Table 4 (panel A) also shows that the significant effects of sex on TG, HDL-C, and diastolic BP, which are abolished by the ratio of visceral-to-peripheral adiposity, are also abolished by indices of lean mass (FFMI or ALMI). However, the application of a stepwise regression analysis, which allows the testing of the contribution of subsets of the independent variables to the overall model (supplementary Table S2) indicates that while visceral-to-peripheral adiposity ratio and lean mass together contribute to 31%, 15 and 28% of variability in TG, HDL-C, and diastolic BP, respectively, the contribution of lean mass per se is small (about 3–5%), and that visceral-to-peripheral adiposity ratio is a more important contributor of variability in TG, HDL-C, and diastolic BP.

**Table 4:** Analysis of the effect of sex (A) and ethnicity (B) on cardiovascular risk markers before adjustments (‘Unadjusted’ row) and after adjustments for adiposity or lean mass indices as covariates.

*A. Effect of sex*	TG	HDL-C	LDL-C	Systolic BP	Diastolic BP
*Unadjusted*	M > W***	M < W**	ns	M > W***	M > W***
*Adjusted for covariates*					
Central adiposity					
Visceral fat (kg)	***	**	**-**	***	***
Subcutaneous (kg)	***	***	**-**	***	***
Visceral/Android	***	**	**-**	***	***
Visceral/Subcutaneous	***	**	**-**	***	***
Peripheral adiposity					
Appendicular (Limb) (kg)	***	***	**-**	***	***
Gynoid (kg)	***	***	**-**	***	***
Central-to-Peripheral adiposity					
Visceral/limb	ns	ns	**-**	***	ns
Subcutaneous/limb	(**)	(*)	**-**	***	(**)
Visceral/Gynoid	ns	ns	**-**	***	ns
Subcutaneous/Gynoid	(**)	(*)	**-**	***	(**)
Lean mass; FFMI or ALMI (kg/m^2^)	ns	ns	**-**	***	ns

*B. Effect of Ethnicity*	TG	HDL-C	LDL-C	Systolic BP	Diastolic BP
*Unadjusted*	I > C***	I < C***	I > C*	ns	ns
*Adjusted for covariates*					
Central adiposity					
Visceral fat (kg)	(**)	***	*	**-**	**-**
Subcutaneous (kg)	***	***	*	**-**	**-**
Visceral/Android	***	***	*	**-**	**-**
Visceral/Subcutaneous	***	***	*	**-**	**-**
Peripheral adiposity					
Appendicular (Limb) (kg)	***	***	**	**-**	**-**
Gynoid (kg)	***	***	**	**-**	**-**
Central-to-Peripheral adiposity					
Visceral/limb	(*)	(**)	ns	**-**	**-**
Subcutaneous/limb	***	***	*	**-**	**-**
Visceral /Gynoid	(*)	(**)	ns	**-**	**-**
Subcutaneous/Gynoid	***	***	*	**-**	**-**
Lean mass; FFMI or ALMI (kg/m^2^)	***	***	*	**-**	**-**

An impact of the covariate in abolishing partially is shown in parenthesis or in abolishing completely as ‘ns’.

*M* Men, *W* Women, *I* Indian, *C* Creole, *ns* not significant, *FFMI* fat-free mass index, *ALMI* appendicular lean mass index.

*p ≤ 0.05; **p ≤ 0.01; ***p ≤ 0.001.

The results presented in Table 4 (B) indicate that the significant effects of ethnicity on blood lipid risk markers (TG, HDL-C, LDL-C) are mostly unaltered after adjusting for the parameters of central adiposity (visceral, abdominal subcutaneous, visceral/android, visceral/subcutaneous), or for peripheral adiposity (limb fat or gynoid fat). By contrast, these effects due to ethnicity are reduced or abolished after adjusting for indices of visceral-to-peripheral adiposity (visceral/gynoid, visceral/limb), but not after adjusting for abdominal subcutaneous-to-peripheral adiposity (subcutaneous/gynoid; subcutaneous/limb). The significant effects of ethnicity on TG, HDL-C, and LDL-C are also not altered after adjustments for the indices of lean mass (FFMI or ALMI).

The significant effects of sex and ethnicity on cardiometabolic health markers are also not altered after adjusting for FMI.

## Discussion

The study presented here in young adult Mauritians demonstrates a less favorable cardiometabolic risk profile in men than in women, and in Indians than in Creoles, which is explained at least in part by a higher visceral adiposity characterized as the ratio of visceral-to-peripheral adiposity rather than by a disproportionate visceral adiposity per se. Furthermore, although lean mass also differed by sex and ethnicity, the results of our analysis indicates that lean mass, in its own rights, has a relatively small impact or no significant impact on the differences due to sex and ethnicity, respectively, on cardiometabolic health markers in this cohort.

Although recruited as apparently healthy subjects, the high prevalence of elevated fasting plasma glucose and high blood pressure (BP) in this population sample (Supplementary Table S1) reflect the outcome of national surveys of the past decades in their characterization of Mauritians as a population with a high predisposition to type 2 diabetes and hypertension [10–17]. In this cohort here of young adults, however, only a few subjects were diagnosed with overt type 2 diabetes and/or hypertension, and sensitivity analysis indicated that their inclusion or exclusion in data analysis has no impact on our findings elaborated and discussed below. In this context, this research outcome can be viewed as one based on an essentially disease-free young adult Mauritian cohort.

### Regional body composition

The results presented here on DXA-derived body composition reinforce previous findings, from studies that assessed body composition in adults and children by isotopic deuterium dilution and bioimpedance analysis, that for the same age, sex, and BMI, Mauritian Indians have more body fat and less lean mass than Creoles [28, 29], and that in young adults of the same sex and WC, Indians have more abdominal fat than Creoles [28]. Using DXA-derived regional body composition analysis, the current study also reveals a more adverse regional pattern of fat distribution characterized by disproportionately higher visceral adiposity in men than in women, and in Indians than in Creoles.

Our results are in line with the well-documented differences due to sex in regional fat distribution in other populations, with women being generally characterized by a preferential accumulation of adiposity in the gluteo-femoral region, whereas men are more prone to abdominal fat deposition [30]. Indeed, subgroup comparisons here also indicate that Mauritian men show higher values than women for the various indices of central-to-peripheral adiposity (android/gynoid, android/limb). Furthermore, the findings here that Indians have disproportionately more visceral adiposity than Creoles are in agreement with some studies conducted in Canada, USA, and UK indicating that migrant populations of South Asian descent have more visceral fat for the same BMI or WC than those of European descents whereas those African descents have less [31–33].

There are, however several studies that have failed to find a higher visceral adiposity in people of South Asian origins, and a recent systematic review and meta-analysis of new and published data has indicated that most studies reported similar levels of visceral fat in South Asians compared with those of white European descent [34]. In addition to low power of single studies and the use of different techniques and approaches to determine visceral adipose tissue, these discrepancies across studies may also be contributed by the way visceral fat is expressed or adjusted. In fact, in our study, ethnic differences in the absolute level of visceral fat (kg), although numerically higher in Indians than in Creoles, did not reach statistical significance. A disproportionately higher visceral adiposity in Indians than in Creoles (as well as due to sex) only became evident when visceral fat was linearly regressed against total fat or trunk fat, or when it is expressed as a fraction of android fat (and hence relative to subcutaneous fat in that abdominal region) or expressed relative to peripheral fat (in the gynoid region or in limbs). These latter analytical approaches may thus be more appropriate in revealing differences in body fat distribution patterns across ethnically diverse populations. Our findings of a disproportionate higher visceral fat in Indians than in Creoles across the entire body fat range studied, including in those with low body fat, as well as in an essentially young and disease-free cohort, would therefore support the contention of Wells [35] that South Asians inherently allocate fat disproportionately to the visceral depot.

### Cardiovascular health markers: sex differences

Sex differences in cardiometabolic risks are generally attributed to a combination of biological and behavioral factors which can be modified by age, ethnic background, and geography [36, 37]. Compared to premenopausal women, men tend to have a less favorable cardiometabolic risk profile in terms of higher fasting blood glucose, TG, LDL-C, and BP. These sex differences have often been shown to be independent of BMI and WC and to persist after adjustments for various lifestyle behaviors (physical activity, smoking). In the present study in young Mauritian adults who were non-smokers and non-regular alcohol consumers, these less favorable cardiometabolic risk factors in men than in women were observed in their serum TG and HDL-C and in BP. The higher TG and low HDL-C in men than in women, could in part be associated with a greater visceral-to-peripheral adiposity ratio in men than in women.

As for the higher BP (and higher incidence of hypertension) in young and middle-aged men than in premenopausal women [36, 37], the explanations generally center upon the protective role of female sex hormones and their receptors, and the role for androgen, possibly through mechanisms that blunt the pressure-natriuresis relation [38]. In our study here in young adults, although both systolic and diastolic BP were higher in men than in women (by about 14 and 7 mm Hg, respectively), only the sex difference in diastolic BP could be related to body fat distribution in that it was abolished after adjusting for visceral-to-peripheral adiposity.

To try to understand why the differences in diastolic BP are more affected after adjustment for the ratio of visceral-to-peripheral adiposity than it is the case for systolic BP, one should first consider that systolic and diastolic BP represent the extremes values of the BP oscillations within each cardiac cycle. However, from a hemodynamic point of view, what is determinant to achieve sodium balance through the pressure-natriuresis mechanism is the mean BP [39, 40], which is the variable used to compute total peripheral resistance. Yet, various factors can affect mean BP, notably renal compression by visceral, retroperitoneal, and renal sinus fat [41, 42]. As diastolic BP is closer to mean BP and thus more closely related to total peripheral resistance (which reflects the state of constriction of small arteries and arterioles), whereas systolic BP is more closely related to the elasticity of the central arteries, one could thus speculate that adjustment for the ratio of visceral-to-peripheral adiposity is more likely to affect diastolic BP than it does on systolic BP.

### Cardiovascular health markers: ethnic differences

While BP tended to be higher in Creoles than in Indians, this ethnic difference in systolic or diastolic BP was, however, marginal and not statistically significant. These results here would seem to be in conflict with the results of national surveys which have consistently reported significantly higher BP in adult Creoles than in Indians, albeit for a population sample that also included middle-aged and older adults [43, 44]. However, our study here in young adult Mauritians that BP is not significantly higher in Creoles than in Indians is consistent with findings from the UK that higher values for BP in people of African ancestry are seen in older, but not in younger age groups [45–47].

Our data indicating a less favorable blood lipid profile (higher TG and lower HDL-C) in Indians than in Creoles in this young adult cohort is also consistent with previous findings from national surveys based on pooled data of Mauritians aged 20–70 years [15–17]. Furthermore, in line with a previous study indicating that this ethnic difference in blood lipid profile could not be explained by abdominal adiposity assessed as waist-to-hip ratio, our study here also indicates that the more adverse lipid profile in Indians than in Creoles is not explained by the ratio of android/gynoid fat (for which waist-to-hip ratio is a proxy), nor by visceral fat or central adiposity indices per se. By contrast, only the ratio of visceral-to-peripheral adiposity could explain, albeit partly, the more adverse lipid profile in Indians than in Creoles. The specificity of visceral fat rather than subcutaneous fat in the abdominal android region in this explanation is underscored by the lack of impact of adjusting for abdominal subcutaneous-to-peripheral adiposity in this ethnic difference in lipid profile.

### Visceral-to-peripheral adiposity ratio vs visceral adiposity per se

While genetics and lifestyle factors could also contribute to the more adverse lipid profile in Indians than in Creoles, the question arises as why it is the visceral-to-peripheral adiposity ratio, rather than visceral fat or visceral/android fat, that explain at least partly the ethnic (as well as sex) differences in cardiometabolic risks. In addressing this question, it is important to consider that the primary adipose tissue compartment which is present throughout the body, i.e. the superficial subcutaneous adipose tissue, constitutes the vast majority of the adipose tissue in the lower body and limbs. Rather than being relatively metabolically inert or benign, these fat depots, which differ from intra-abdominal and visceral adipose tissue by their developmental and functional characteristics, may actively protect against risks for cardiovascular diseases [48–50], namely (i) by acting as a ‘protective metabolic sink’ capable of metabolizing and storing (and hence buffer against) excess circulating free fatty acids, and (ii) by releasing predominantly anti-inflammatory cytokines. In fact, lower body adiposity, which comprises mostly superficial subcutaneous adipose tissue in the gluteal, femoral, and thigh regions, has been shown to be independently associated with a healthier cardiovascular risk profile in several ethnic groups [51–56], including in Asian Indians [56]. The protective effects of peripheral (superficial) adipose tissue therefore contrast sharply with the hazardous effects of visceral adipose tissue and deep abdominal subcutaneous adipose tissue through the release FFAs and pro-inflammatory cytokines that impair hepatic (and lipid) metabolism [19]. Consequently, a ratio of visceral-to-peripheral adiposity, by reflecting the opposing effects of hazardous fat (visceral) versus protective fat (superficial subcutaneous) best defines the net susceptibility to atherogenic disease risk. This would imply that two individuals, one with low visceral and peripheral fat, and the other with high visceral and peripheral fat, would have the same risk for high TG or low HDL levels.

### Study limitations and strengths

This is the first experimental study that has applied DXA technology to investigate body composition in Mauritians, and specifically here into the effects of sex and ethnicity on the relationship between body composition and cardiometabolic risks in this population with a high propensity for type 2 diabetes and cardiovascular diseases. A main limitation of this study is that the population sample is relatively small, particularly among men of Creole ethnicity. However, this is to some extent compensated by the selection of subjects as young adults, essentially without diabetes and disease-free, all non-smokers, non-regular alcohol consumers, and non-physically active; thereby obviating the need to adjust for these well-known confounding clinical and lifestyle factors for cardiovascular risks, and hence the need for much large sample size. Furthermore, the large range of BMI and WC (and hence body composition parameters) in all subgroups satisfied conditions for appropriate analytical procedure centered on regression models. Another limitation is the validity of assessing visceral fat by DXA, which was not measured but estimated according to an algorithm which was developed on US population. However, validation of DXA-derived estimation of visceral fat against gold-standard MRI, have indicated that DXA, a relatively convenient, inexpensive, and safe method with minimum radiation dosage, can be a reliable technique for assessment of visceral fat in several other populations, including in Asian populations [57–60].

## Conclusions

This study in young (disease-free) adult Mauritians demonstrates a disproportionately higher visceral

compared to peripheral adiposity in Indians than in Creoles across a wide range of body fat, and is hence in support of the contention that South Asians allocate fat disproportionately to the visceral depot. It also suggests that differential effects of sex and ethnicity on cardiovascular risk profile are better explained by differences in the visceral-to-peripheral adiposity ratio than by a disproportionate visceral adiposity *per se;* the former possibly reflecting the ratio of hazardous (visceral) adiposity and protective (peripheral) superficial subcutaneous adiposity. Further studies are warranted to validate the visceral-to-peripheral adiposity ratio as a critical body fat distribution pattern in the pathogenesis of cardiovascular diseases in ethnically diverse populations.

### Supplementary information

Supplementary Tables S1 and S2

## Data Availability

The datasets analyzed during the current study are available from the corresponding author on reasonable request.
