# Supplementary material for: Visceral-to-peripheral adiposity ratio: a critical determinant of sex and ethnic differences in cardiovascular risks among Asian Indians and African Creoles in Mauritius
Source: Int J Obes (Lond). 2024 Apr 13;48(8):1092–102. doi: 10.1038/s41366-024-01517-3 (PMC11281908; doi:10.1038/s41366-024-01517-3)
Supplement: Supplementary file 1 — Supplementary Tables S1 and S2 [file 41366_2024_1517_MOESM1_ESM.docx]

**Supplementary material *Table S1***

**Percentage (%) of subjects (n=189) with cardiometabolic risks**

**% of subjects**

***Glycemic profile***

**Glucose** (> 5.5 mmol/L) 12

- Prediabetes (5.6 – 6.9) 10
- Diabetes (≥ 7.0) 2

**HbA1c** (> 5.6%) 44

- Prediabetes (5.7–6.4%) 41
- Diabetes (≥ 6.5%) 3

***Lipid profile***

Triglycerides (> 2.0 mmol/L) 8

Cholesterol (mmol/L)

- Total (> 5.2) 19
- HDL (< 0.9) 4
- LDL ( > 4.1) 4

***Blood Pressure (BP)***

**Systolic BP** (mm Hg)

- Stage 1 (130-139) 3
- Stage 2 (≥ 140 ) 4

**Diastolic BP** (mm Hg)

- Stage 1 (80-89) 23
- Stage 2 (≥ 90 ) 9

**Supplementary material *Table S2***

Stepwise linear regression of cardiometabolic health parameters that differed by sex (TG, HDL-C, Diastolic BP) vs independent (body composition) predictor variables

**TG HDL-C Diastolic BP**

***r ^2^ r ^2^ r ^2^***

**Independent**

**predictor variables**

step 1 0 0 0

step 2 Visceral/Gynoid 0.28*** - 0.21***

Visceral/Limb - 0.12*** -

step 3 Fat-free mass Index 0.31** 0.15*** 0.26***

***% Contribution to variability***

- Visceral-to-peripheral

adiposity ratio 28% 12% 21%

- Fat-free mass index 3% 3% 5%

Level of statistical significance of r^2^ values: *** = p<0.001; ** = p<0.01; * = p<0.05
